# Non-Lethal Blasts can Generate Cavitation in Cerebrospinal Fluid While Severe Helmeted Impacts Cannot: A Novel Mechanism for Blast Brain Injury

**DOI:** 10.3389/fbioe.2022.808113

**Published:** 2022-07-07

**Authors:** Xiancheng Yu, Thuy-Tien Nguyen, Tianchi Wu, Mazdak Ghajari

**Affiliations:** ^1^ HEAD lab, Dyson School of Design Engineering, Imperial College London, London, United Kingdom; ^2^ Centre for Blast Injury Studies, Imperial College London, London, United Kingdom; ^3^ Department of Bioengineering, Imperial College London, London, United Kingdom

**Keywords:** cavitation, cerebrospinal fluid, blast TBI, traumatic brain injury, impact TBI

## Abstract

Cerebrospinal fluid (CSF) cavitation is a likely physical mechanism for producing traumatic brain injury (TBI) under mechanical loading. In this study, we investigated CSF cavitation under blasts and helmeted impacts which represented loadings in battlefield and road traffic/sports collisions. We first predicted the human head response under the blasts and impacts using computational modelling and found that the blasts can produce much lower negative pressure at the contrecoup CSF region than the impacts. Further analysis showed that the pressure waves transmitting through the skull and soft tissue are responsible for producing the negative pressure at the contrecoup region. Based on this mechanism, we hypothesised that blast, and not impact, can produce CSF cavitation. To test this hypothesis, we developed a one-dimensional simplified surrogate model of the head and exposed it to both blasts and impacts. The test results confirmed the hypothesis and computational modelling of the tests validated the proposed mechanism. These findings have important implications for prevention and diagnosis of blast TBI.

## 1 Introduction

Blast-induced traumatic brain injury (BTBI) has been a prevalent injury in recent military conflicts due to the vast use of improvised explosive devices (IEDs) (Rosenfeld et al., 2013). However, the role of blast wave in producing brain injury is not well understood. This has left a gap in our approach to preventing BTBI. Traditional head protection equipment, such as helmets, are designed to protect the head against impact and ballistic loadings, such as bullets and shrapnel penetration and impacts with objects and ground after blast exposure. However, their performance in mitigating injuries induced by blast wave is largely unknown. A previous neuropathological analysis of brain tissue from post-mortem cases of blast and impact induced TBIs has shown that blast exposure produces unique patterns of damage (astroglial scarring) that are distinct from those associated with impact exposure (Shively et al., 2016). The damage was mainly located at the boundaries between the brain tissue and fluid in cases with blast exposure. However, cases with impact exposure alone did not show the same pattern of damage.

A likely physical mechanism for such damage at the brain/fluid interfaces is cerebrospinal fluid (CSF) cavitation. Fluid cavitation is the formation and collapsing of cavities or bubbles in a liquid. It occurs when the local pressure in the liquid is reduced below a certain value called the vapor pressure. The collapse of the cavitation bubbles produces local shock waves and high-speed micro-jets, which can elevate the surrounding temperature to 6000 K (Franck, 2017). Cavitation-induced micro-jets have the capability to damage even the strongest man-made materials (Brennen, 2014; Franck, 2017). Cavitation in CSF has been suggested as a possible mechanism of traumatic brain injury (TBI) (Galloway, 1954; Gross, 1958; Lubock and Goldsmith, 1980; Nakagawa et al., 2011; Goeller et al., 2012; Canchi et al., 2017; Salzar et al., 2017; Bustamante and Cronin, 2019). However, the onset and mechanism of cavitation in the CSF during impact and blast exposure remain unclear. This restricts the diagnosis, mitigation and prevention of the possible cavitation damage in the human head.

In this study, we investigated the CSF cavitation under both impact and blast loadings. First, we used a finite element (FE) model of the human head to simulate the head response to blast and impact loadings. Informed by the simulation results, we proposed a novel mechanism for the formation and collapse of cavitation in the human head. Then, based on this mechanism, we hypothesised that CSF cavitation can be induced by blast, but not helmeted impacts typical in road traffic and sporting collisions. Next, we tested this hypothesis by developing a one-dimensional surrogate model and exposing it to both blast and impact loadings. Finally, we simulated the tests using an FE model of the surrogate to validate the test results and explain the cavitation mechanism.

## 2 Methods

### 2.1 The FE Models and Simulation Methods

**FIGURE 1:**
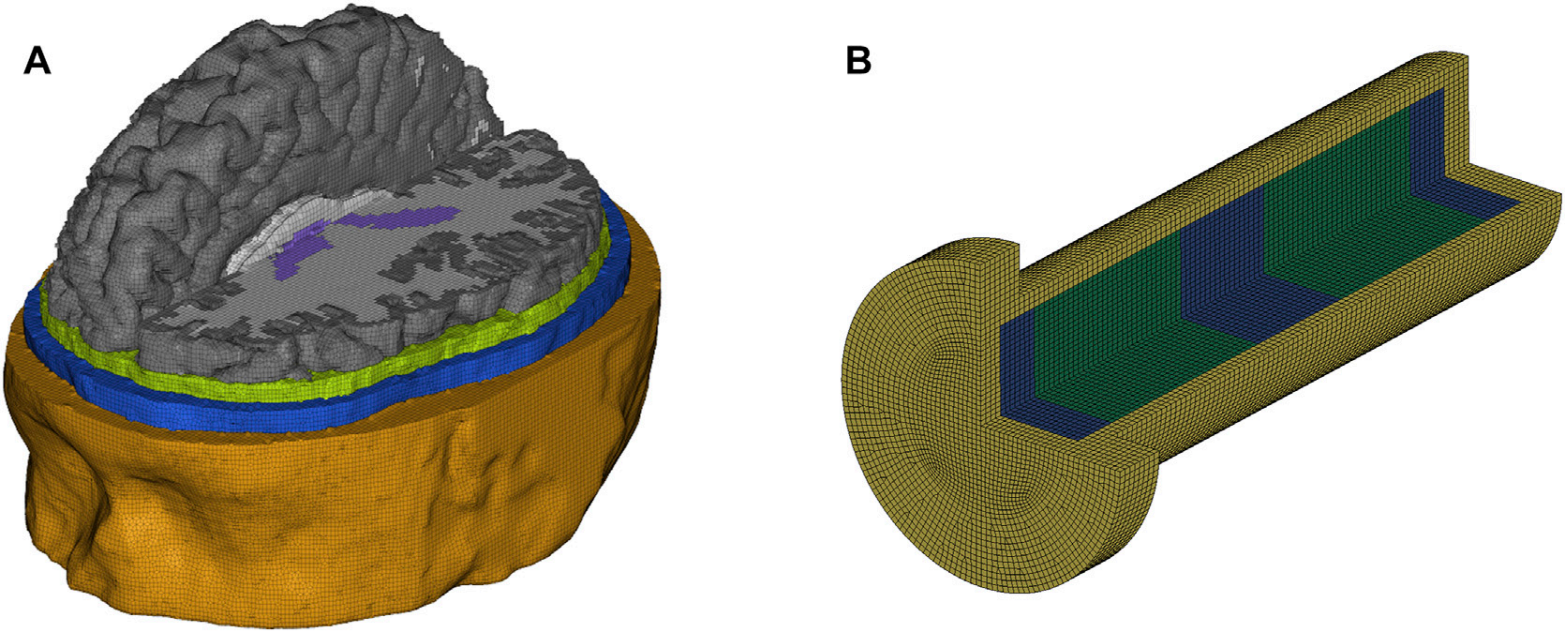
FE models of **(A)** human head and **(B)** 1D surrogate model.

The three-dimensional (3D) human head FE model used in this paper was developed in a previous study (Figure 1A) (Ghajari et al., 2017). The head model incorporates detailed anatomy of 11 tissues, including skin, skull, CSF, ventricles, white matter, grey matter etc. The material models and properties of the tissues were taken from our previous study (Yu et al., 2020). The 1D surrogate FE model was developed in accordance with the 1D surrogate model dimensions in this study (Figure 1B). The material models and properties of the CSF surrogate (distilled water) and brain (agarose gel) surrogate were identical to the properties of the CSF and brain in the 3D human head FE model. The skull surrogate (acrylic) was modelled with a hyperviscoelastic material model with properties reported in a previous study (Bustamante et al., 2018). The pressure response of the human head FE model was validated against the experimental data from a previous study (Nahum et al., 1977), which conducted impact tests on cadaver heads and measured the impact force and intracranial pressures (Supplementary Materials). In addition, the prediction of the head FE model for brain deformation has been validated against recent cadaver experiments, where the post-mortem human subject heads were subjected to well-controlled rotations (Alshareef et al., 2018). The validation detailed can be found in our previous studies (Fahlstedt et al., 2021; Zimmerman et al., 2021).

The blast wave and its interaction with the head were modelled with the prescribed inflow and ALE (Arbitrary Lagrangian-Eulerian) methods in LS-DYNA nonlinear hydro-code (LSTC, 2007). A multi-size air mesh domain was used to avoid the blast wave reflection from the air domain boundaries without increasing the computational time significantly. The modelling method can be found in our previous study (Yu and Ghajari, 2019). In the impact simulations, the head was launched towards a 20 mm expanded polystyrene (EPS) foam (density: 60 kg/m3), where one side of the EPS foam was fixed. The material model and properties of the EPS foam were taken from (Ghajari et al., 2011).

Using the human head FE model, we conducted two simulations: a non-lethal blast and a typical road traffic/sports impact. For blast, the overpressure was 2.19bar and positive duration was 1.43 ms. For impact, we simulated a 4 m/s impact on a 20 mm thick layer of EPS foam, similar to most padded impacts, e.g., in bicycle accidents.

### 2.2 The 1D Surrogate Model and Test Methods

**FIGURE 2:**
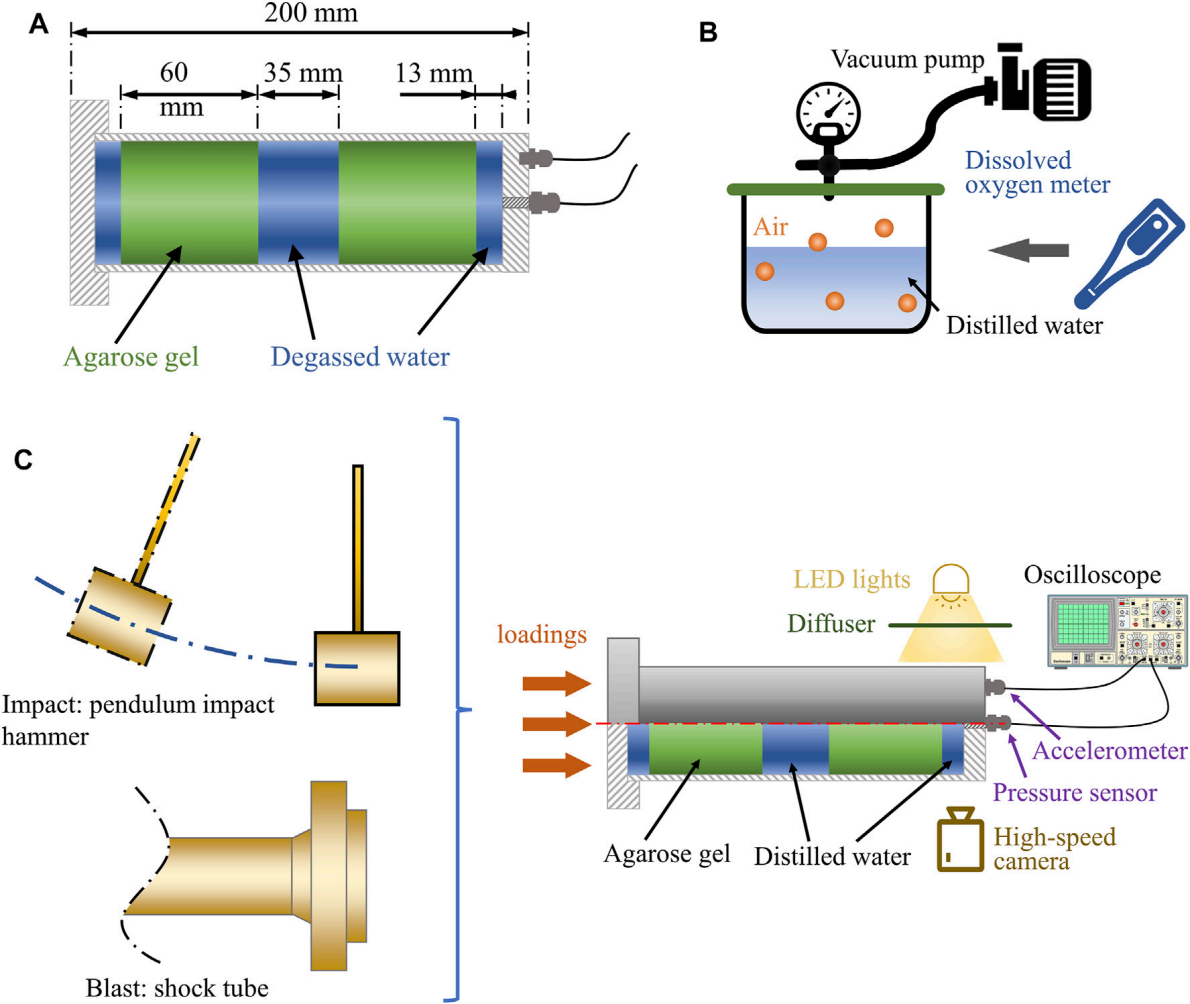
**(A)** The dimension of the 1D surrogate model. **(B)** The water degasification. **(C)** The set-up of the blast and impact tests.

We simplified the human head as a one-dimensional (1D) surrogate model (Figure 2A) because the phenomena discussed here are related to wave propagation along one dimension. A similar approach has been used previously to study cavitation produced by under-water explosion (Schiffer and Tagarielli, 2012). As the inner diameter of the shock tube is 59 mm, the outer and inner diameter of the acrylic tube were chosen to be 60 and 50 mm. This was to ensure the surrogate model experienced a uniform blast wave loading. The total length of the model was 200 mm, nearly equal to the length of the 50^th^ percentile male human head. The volume was filled with two 60 mm thick disks of agarose gel (representing the brain tissue), which were separated from the skull with a 13 mm thick layer of distilled water (representing cortical CSF) and from each other with a 35 mm layer of distilled water (representing ventricular CSF). These dimensions were based on measurements of the human head FE model (Figure 1A). The thickness of the cortical CSF was based on the distance between the skull and cortex, considering both sulci and gyri. The thickness of the ventricular CSF was based on the anterior-posterior length of the third ventricle.

We used surrogate materials whose acoustic impedances were similar to the human tissues to ensure that the pressure wave transmission and reflection in the surrogate materials were similar to their corresponding tissues. The human skull was modelled with a transparent acrylic tube, sealed with leak-proof threaded caps at both ends. The brain tissue was modelled with agarose gel (0.65% concentration), as suggested in a previous study (Deepthi et al., 2010). The cortical and ventricular CSF were modelled with distilled water. It should be noted that CSF in biological conditions contains dissolved air. Previous studies used degassed distilled water to model CSF (Goeller et al., 2012; Hong et al., 2016; Bustamante and Cronin, 2019). This probably led to underestimation of CSF cavitation as CSF can carry dissolved air. Here, we measured and controlled the dissolved air in the distilled water to the level determined in a previous study on human subjects (Zaharchuk et al., 2006) (Figure 2B).

Figure 2C shows the set-up of the blast and impact tests. An accelerometer (PCB® model 353B14) and a pressure sensor (Dytran® model 2300V3) were mounted on the rear cap of the surrogate model to measure acceleration and pressure-time histories. The pressure sensor was mounted flush to the inner wall of the rear cap, contacting the water, where initial cavitation was expected. Inside this pressure sensor, there is an additional integral accelerometer with a seismic mass and a quartz crystal, which can produce a signal opposite to the applied pressure to cancel the mechanical motion by acceleration or shock on the output (Nguyen, 2016). Its acceleration sensitivity in axial direction is 0.0069 kPa/g. In this study, the maximum acceleration in blast test was around 1200 g, resulting in a negligible 8.28 kPa error in pressure. The motion of the head surrogate was restricted by guided steel rail, only allowing motion along the shock tube axis to remove the effect of transverse load on pressure sensor recording. Data from both sensors were sampled by an oscilloscope at 50 MHz. A high-speed camera was used to capture 79,000 frames-per-second recordings of the bubbles formation and collapse in the contrecoup CSF region.

The blast tests were conducted with a 59 mm-diameter shock tube system, as described in (Nguyen et al., 2019). The details of the blast test method has been introduced in our recent study (Yu et al., 2022). The surrogate model was placed at 3.5 mm from the outlet of the shock tube. This distance ensures that the head surrogate experiences nearly planar wave while it does not obstruct the air flow dissipation after the impingement. The characterization of this apparatus has shown that the blast overpressure and waveform within 40 mm from the shock tube outlet remain approximately the same, and they are similar to the classic Friedlander waveform.

The impact tests were conducted with a pendulum impact hammer (3 kg mass). We attached EPS foams with 50–70 kg/m^3^ density and different thicknesses onto the front cap of the surrogate model to generate acceleration-time histories similar to those seen in falls, road traffic and sporting collisions, where helmets are worn (Ghajari et al., 2017). Table 1 shows the parameters of the blast and impact tests. The three blast loadings reported in this study were characterised as non-lethal (no risk of pulmonary or head injury) (Rafaels et al., 2011; Panzer et al., 2012). The three impact loadings can generate 100–300 g accelerations on the surrogate model, which were categorised as moderate to severe head impacts (Kleinberger et al., 1998; Golfo et al., 2019). To test the repeatability of the tests, impact 2 and blast 3 were repeated three times. We calculated the coefficient of variation for the peak acceleration and peak negative contrecoup pressure among the repeats. The results showed that the coefficients of variation for both peak acceleration (impact: 1.5%; blast: 3.4%) and peak negative contrecoup pressure (impact: 1.2%; blast: 3.5%) were low, indicating good repeatability of both the impact and blast test.

**TABLE 1:** Blast and impact test conditions.

Blast tests	Overpressure (bar)	Positive duration (ms)
Blast 1	1.415	0.676
Blast 2	1.832	1.015
Blast 3	2.271	1.434
**Impact tests**	**Impactor speed (m/s)**	**Paddings**
Impact 1	5.68	40 mm EPS 50
Impact 2	4.90	30 mm EPS 70
Impact 3	4.90	20 mm EPS 50

## 3 Results and Discussion

### 3.1 Human Head Simulations Show That Blast can Induce Lower Contrecoup Pressure Than Impact

**FIGURE 3:**
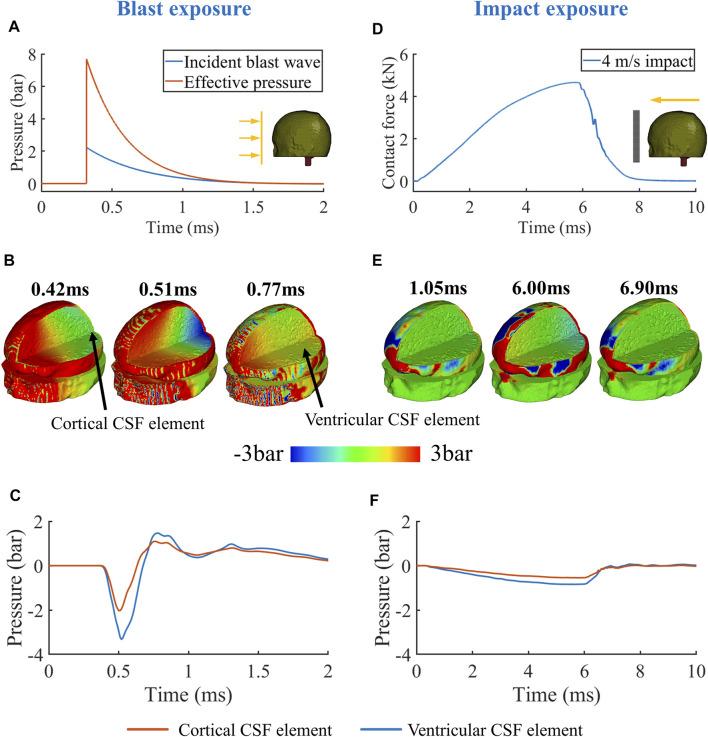
**(A)** Typical blast (pressure wave) and **(D)** impact loadings (contact force). **(B,E)** Pressure contour. **(C,F)** Contrecoup pressure history.

The key difference between the blast and impact was the rising edge of the loading profiles (Figure 3A&D). For blast, the peak of the effective pressure, which is the sum of the incident and reflected pressures, was 7.8bar. The instant rise of the pressure from 0 to peak produced a compressive pressure wave with high magnitude and sharp rise in the scalp, which transmitted into the skull, CSF and brain tissue. Figure 3B shows that at 0.42 ms the pressure wave in the skull (outer wave) transmitted faster than that in the brain (inner wave) due to the higher acoustic speed in the skull than in the brain. The outer wave arrived at the contrecoup site before the inner wave and accelerated the skull at this region. This initiated a relative velocity at the skull/CSF interface, which in turn produced a tensile wave propagating back to the CSF and brain. At 0.51 ms, the tensile wave created a negative pressure region at the contrecoup region, while the inner wave was still propagating towards this region (Figure 3B).

The inner wave continued its propagation, interacted with the tensile wave, and finally canceled the negative pressure in the contrecoup region. At 0.77 ms, the pressure at this region was positive (Figure 3B). Figure 3C shows the pressure time histories of two elements, one in the contrecoup cortical CSF and one in the contrecoup ventricular CSF (rear end of the lateral ventricle). The plots show that the pressure was first decreased by the tensile wave to below -2bar and then returned to positive by the inner wave. As the inner wave reached the ventricle first, pressure at the contrecoup ventricular CSF element started its return to positive values before the contrecoup cortical CSF element.

Unlike blast, the simulated impact applied a bell-shaped contact force on the head, with a gentle rising edge and much longer duration before reaching its peak (Figure 3D). As a result, the magnitude of the outer wave was not as large as that produced by the blast (Figure 3E). With the increase of the contact force over time, higher-magnitude outer and inner waves were generated. However, it took only 0.07 ms for the outer wave and 0.13 ms for the inner wave to transmit from the loading site to the contrecoup site. Thus, during the 5.7 ms time of contact force rising from zero to peak, the outer and inner waves had transmitted inside the head around 44–81 times. This indicates that the tensile wave in the contrecoup CSF, which was generated by the outer wave, had interacted with the inner wave many times before reaching its peak (Figure 3E). As a result, the pressure histories of the contrecoup cortical CSF and ventricular CSF elements had a shape similar to the shape of the contact force (Figure 3F). When the contact force reached its peak, the pressures at the contrecoup CSF and lateral ventricle also reached their peaks. The pressure slowly returned to 0 as the contact force declined, resulting in a declining relative velocity at the contrecoup skull/CSF interface.

Based on the above observations of wave transmission in the human head under blast, we propose a new mechanism for CSF cavitation: the outer wave propagating in the skull produces a negative pressure zone in CSF at the contrecoup region, which is later cancelled by the slower inner wave propagating in the soft tissue. In addition, informed by the above observations of contrecoup pressures under blast and impact, we hypothesised that cavitation can occur in the CSF during typical blast exposures but not typical impact exposures.

### 3.2 Surrogate Model Tests Confirm That Blast, and not Impact, can Produce Cerebrospinal Fluid Cavitation

**FIGURE 4:**
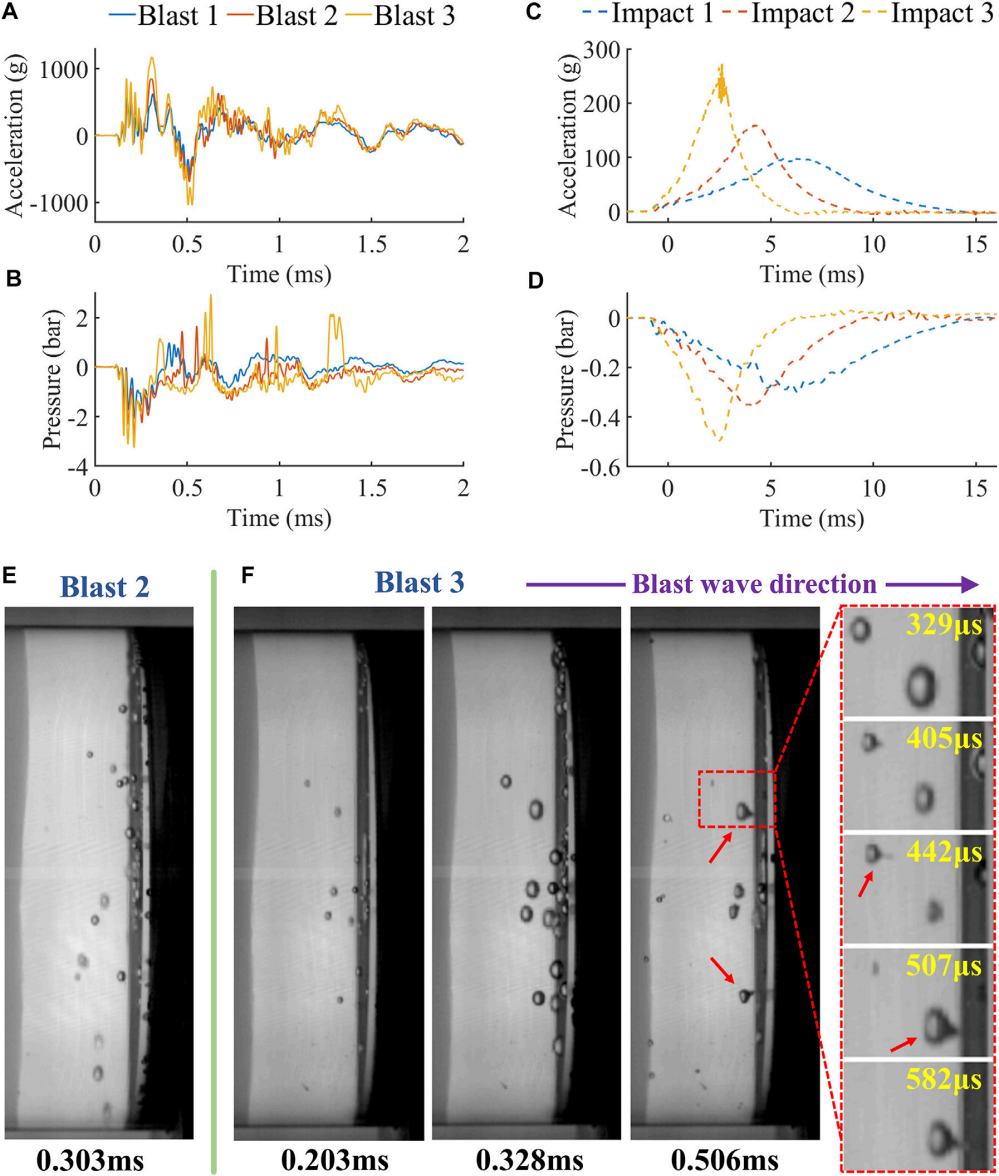
The acceleration and pressure histories of **(A,B)** blast tests and **(C,D)** impact tests. **(E)** The high-speed video footages of blast tests and the formation of micro-jets (red arrow).

Figure 4 shows the test results of the surrogate model under blasts and impacts. First, we compared the acceleration and pressure histories of the tests (Figures 4A–D). The accelerations produced by the blasts were nearly three folds higher than those produced by the impacts, with the largest peak acceleration reaching values as high as 1177 g at a short time of 0.31 ms. This peak acceleration is at the low-moderate level of peak accelerations produced by blast exposures, recorded in previous blast tests on a porcine head (358–3845 g) (Shridharani et al., 2012) and human surrogate (3500 g) (Goeller et al., 2012). The accelerations produced by impacts represent the typical head collisions during road traffic/sporing accidents, with a bell shape, peak values between 100-300 g and durations between 5-15 ms (Figure 4C). Impact 3 is characterised as a severe impact as the acceleration corresponds to a 55.4% probability of a severe injury (complex facial fractures, exposure or loss of brain tissue, small epidural or subdural hematoma) and a 88.5% probability of serious injury (different fractures, loss of scalp, bruises to the cerebellum) (Kleinberger et al., 1998; Golfo et al., 2019).

The largest difference between the blast and impact was the rise time and peak of the acceleration histories. Blast-induced accelerations reached their first peaks at least 60–130 times faster than impact-induced accelerations. This large difference produced the different type of contrecoup pressure histories in blast and impact tests shown in Figure 4B&D. Similar to the acceleration histories, the contrecoup pressure took much less time to reach its peak under blast than impact (70–148 times faster). Increasing the blast intensity increased the acceleration and reduced the negative pressure at the contrecoup CSF. Similarly, higher impact velocity produced lower negative pressure peaks.

The analysis of the high-speed video footage of the tests showed that fluid cavitation occurred in blast 2 and 3 only (Figure 4E&F). Although the exact cavitation threshold for the CSF surrogate is unknown, the cavitation formation requires negative pressures of -1bar or lower (Galloway, 1954). Both blast 2 and 3 tests led to large negative pressures (Figure 4B), exceeding the cavitation threshold. In impact tests, the lowest pressure (-0.497bar in impact 3, Figure 4D) was far from -1bar, resulting in no cavitation. Figure 4E&F (at 0.328 ms) show the maximum number of bubbles in blast 2 and 3, respectively. The comparison indicates that larger negative pressure in blast 3 produced larger amount and size of bubbles than those in blast 2.

Moreover, we observed micro-jets formation in blast 3 (see the blast 3 video in the supplementary materials). The sequential video footage (zoom-in region, Figure 4F) shows the process of a micro-jet formation following the asymmetric collapse of a bubble. The bubbles were compressed by the inner pressure wave approaching them from the left side, which led to their collapse and generation of micro-jets in the travelling direction of the inner pressure wave.

We did not observe any fluid cavitation in the impact tests. This is different to a recent study, which showed that impact can induce CSF cavitation (Lang et al., 2021). It should be noted that the impact was generated by dropping a surogate head onto the relatively rigid ground. Such blunt impacts produce accelerations with instant rising edge, which is similar to that induced by blast. In fact, blunt impacts have been used to mimic blast loading to study fluid cavitation (Canchi et al., 2017; Bustamante and Cronin, 2019). However, the impacts in our study represent loadings in road traffic and sporting collisions, where helmets are worn and blunt impacts are avoided.

### 3.3 FE Modelling of the Surrogate Validate the Proposed Mechanism for Blast Induced Cerebrospinal fluid Cavitation

**FIGURE 5:**
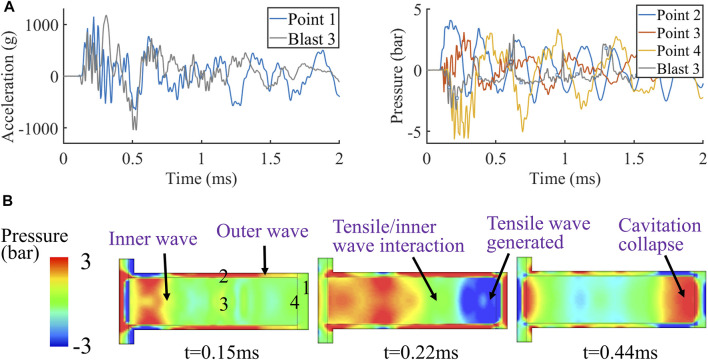
Simulation results of the blast 3 test: **(A)** acceleration and contrecoup pressure histories and **(B)** pressure contour.

Next, we used FE simulations to explore whether our proposed CSF cavitation mechanism can explain the observed cavitation under blast loading. We focused on blast 3 test, where largest number of cavitation bubbles were observed. To validate the FE model, we compared model predictions with the experimental data. Figure 5A shows the acceleration at the contrecoup skull (point 1 in Figure 5B) and pressures at the middle of the skull, middle ventricle and contrecoup CSF (points 2, 3, and 4 respectively in Figure 5B). The predicted acceleration at point 1 has a peak similar to the experimental data. In addition, the oscillations of the predicted and experimental acceleration curves correlate well. The predicted pressure at the contrecoup CSF (point 4) also shows good agreement with the experimental data. The oscillation patterns of the pressure curves from test and simulation are quite similar, with the negative pressure peak occurring at roughly the same time. However, the value of the predicted negative pressure is lower than that in the experiment. This is because when fluid cavitates, the pressure will not be further dropped, but kept at a level, called cavitation threshold or cut-off pressure (Bustamante et al., 2018). Previous computational studies have used different cut-off pressures for the CSF material (Panzer et al., 2012; Zhou et al., 2018; Yu et al., 2020; Yu and Ghajari, 2022). As the cavitation threshold of the CSF surrogate in this study was unknown, we did not include a cut-off pressure in the CSF material model, which led to further drop of the predicted negative pressure compared to the experiments. Overall, we found good agreements between the computational and experimental data, particularly in terms of the timing of the key events, such as the peak acceleration and negative pressure.

Then, we used the predicted pressure contour in the simulation to explore the validity of the outer and inner wave mechanism for blast induced CSF cavitation. A comparison between pressure at the middle skull (point 2) and middle ventricle (point 3) indicates that the outer wave travels faster than the inner wave (Figure 5B). At 0.15 ms, the outer wave has already arrived at the contrecoup skull while the inner wave has just started propagating through the fluid. At 0.22 ms, the outer wave has produced a tensile wave in the fluid, creating a negative pressure zone at the contrecoup area, where cavitation bubbles were observed. The predicted time of cavitation onset correlates well with the video footage of blast 3 (Figure 4F). At this time, the inner wave front has also started interacting with the tensile wave. Finally, at 0.44 ms, the contrecoup pressure is brought to positive by the inner wave, which leads to the collapse of the cavitation bubbles. The predicted timing agrees well with the time of bubbles collapsing in the experiment.

This analysis shows that the duration of the negative pressure at the contrecoup CSF is mainly dependent on the time difference between the outer and inner waves reaching the contrecoup site. A smaller head size will reduce this time difference, which results in a shorter duration of negative pressure at the contrecoup CSF. Therefore, there may be a potential drawback of translating the results of blast experiments on small animals to the human. For example, the breadth of the rat skull is less than 20 mm (Gefen et al., 2003). This small size leads to a very short time difference between the outer and inner waves arriving at the opposite side of the skull, leading to a negligible negative pressure at the contrecoup CSF and very low possibility of producing cavitation. Hence, it may not be possible to produce cavitation in rodent CSF using the same blast intensity expected in the field. This implies that the scaling of the loading conditions may be necessary when using rodents to model human neuropathology under blast loading, a concept that is well known for acceleration-deceleration TBI (Rafaels et al., 2011; Jean et al., 2014).

## 4 Conclusions and Limitations

In summary, we have shown that CSF cavitation can occur under non-lethal blast loadings but not under severe helmeted impact loadings that are typical in falls, sporting and road traffic collisions. In addition, we found that there is a relationship between the peak of the blast pressure wave and cavitation formation and size, with higher-intensity blasts producing more and larger cavitation bubbles. These analyses were allowed by using a 3D finite element model of the human head and a new 1D physical head surrogate model. Our approach also allowed us to explain the mechanisms of cavitation formation and collapse under blast as follows: 1) the blast induced pressure wave in the skull (outer wave) reaches the contrecoup site before the pressure wave in the brain tissue (inner wave), and it initiates a tensile wave in the CSF, which reduces the contrecoup CSF pressure and produces cavitation bubbles; 2) when the inner wave reaches this region, it collapses the cavitation bubbles asymmetrically, leading to the generation of micro-jects. Impact loading cannot produce cavitation due to the smaller pressure peaks and slow rise of the pressure. Our results may explain the neuropathological observations in human cases of blast and non-blast TBI, where damage has been seen at the CSF/tissue interface in the blast cases only (Shively et al., 2016).

Our study has several limitations. First, we simplified the 3D human head into a 1D surrogate model. The 1D surrogate was useful in explaining the key mechanism of CSF cavitation, based on the 1D transmission of outer and inner wave. As the 3D geometry skull reduces the time difference between outer and inner wave reaching the contrecoup region and attenuate the outer wave, human head may experience less severe CSF cavitation than that observed in the surrogate model when exposed to the same blast loading. Future work should extend this model to a 3D head surrogate model and use a larger shock tube in order to investigate the effects of head geometry. This will also allow for investigating the effects of protective equipment, such as combat helmet and goggles. Secondly, in our surrogate model, the CSF surrogate was isolated by the two brain surrogate discs. However, in the human head, CSF is distributed in a more complex pattern, which can affect the location and likelihood of cavitation. For instance, CSF fills the space between the two cerebral hemispheres, which can be a potential path for transmitting part of inner pressure wave to the third ventricle and contrecoup region under frontal blasts. Although the acoustic impedance of the brain and CSF are similar, the distribution of CSF may still influence the occurrence and severity of cavitation, which requires further investigation. Thirdly, the blast waves used in this study had relatively short positive phase durations, which is due to the capacity of the shock tube. Free field blast waves usually have longer positive phase durations, whose effect on cavitation requires further investigation. Our results from physical and FE models however suggest that formation of cavitation is likely to be independent from the positive duration. Finally, we used distilled water as CSF surrogate, but real CSF contains proteins, cells and glucose, which may reduce the cavitation threshold. Future work may use real CSF to predict CSF cavitation more accurately.

Our study suggests that mitigating the risk of cavitation may need to be considered in the evaluation of combat helmets. In addition, our study warrants further investigation into the suitability of using small animals exposed to blast conditions reported in the field to study neuropathology in human. Finally, since the cavitation injury is likely to be confined to interfaces, its diagnosis would require using advanced imaging techniques that allow us to map changes at the CSF/tissue interfaces, which are likely to be missed when using normal CT and MRI scans.

## Data Availability

The original contributions presented in the study are included in the article/Supplementary Material, further inquiries can be directed to the corresponding author.

## Supplementary Material

Supplementary Figure S1
**(A)** Simulation condition to replicate the Nahum’s experiment (Nahum et al., 1977). **(B)** Location of pressure measurements in simulation for comparison with Nahum’s experimental data.

Supplementary Figure S2 Comparison of **(A)** impact force time histories, **(B)** ICP at frontal region, **(C)** ICP at parietal, **(D)** ICP at occipital region 1, **(E)** ICP at occipital region 2 and **(F)** ICP at posterior fossa region.

Supplementary Table S1 Impactor dimension and material properties.
