# Supplementary material for: Non-Lethal Blasts can Generate Cavitation in Cerebrospinal Fluid While Severe Helmeted Impacts Cannot: A Novel Mechanism for Blast Brain Injury
Source: Front Bioeng Biotechnol. 2022 Jul 7;10:808113. doi: 10.3389/fbioe.2022.808113 (PMC9302597; doi:10.3389/fbioe.2022.808113)
Supplement: Supplementary file 2 [file DataSheet1.docx]

**Supplementary materials**

**Validation of the 3D human head FE model**

The 3D human head finite element model used in this paper was developed in a previous study (Ghajari et al., 2017). Here, we validated this model against the intracranial pressure (ICP) time histories, measured by Nahum et al. (Nahum et al., 1977). In Nahum’s study, impact tests on cadaver heads were conducted, using different padded impactors. Nahum et al., (Nahum et al., 1977) provided detailed head response for experiment 37, including impact force and ICP at various locations. We reconstructed the experiment 37 with the 3D human head model. The direction of the impact was 45° with respect to the Frankfort anatomical plane, as shown in Figure S1a.

To replicate the impact condition, we kept the impactor weight (5.59kg) and varied the impact velocity, impactor geometry and padding materials to conduct simulations. After each simulation, the predicted impact force time history was compared with the impact force time history measured from experiment 37. It was found that 4.2m/s impact speed with appropriate impactor configurations best replicate Nahum’s experimental impact force time histories. The impactor dimensions and material properties are shown in Table S1. We compared the impact force time histories from the simulation and Nahum’s experiment in Figure S2a, which showed good agreement. The simulation predicted peak impact force was slightly lower (5.9%) than the experimentally measured value. The time for the impact force rising from zero to the peak were similar.

Next, we compared the ICP measured in different regions and compared them with Nahum’s experimental data. Figure S1b shows the locations of the ICP measurement, including the frontal, parietal, occipital 1, occipital 2 and posterior fossa. For each location, the ICP curve was averaged from 20 elements. Figure S2b-f shows the ICP comparisons between simulation and experiments. Overall, the simulation produced close peak ICPs at all locations (less than 11.5% difference). ICP curves from simulation started rising and reaching their peaks slightly earlier than the experimental data. This is probably due to the simplification of dipole in our head FE model. In the FE model, we simplified the skull as a rigid material, ignoring the dipole structure. In real human head, the dipole may delay the pressure wave transmission from the impact site to the intracranial space. Overall, the head FE model produced reasonably close pressure response to Nahum’s experiment.

Table S1 Impactor dimension and material properties

| **Part** | **Diameter (mm)** | **Thickness (mm)** | **Density (kg/m^3^)** | **Elastic modulus**  **(GPa)** | **Poisson’s rate** |
| --- | --- | --- | --- | --- | --- |
| Rigid mass | 50 | 5 | 570300 | 200 | 0.3 |
| Padding | 50 | 15 | 1000 | 0.01 | 0.16 |


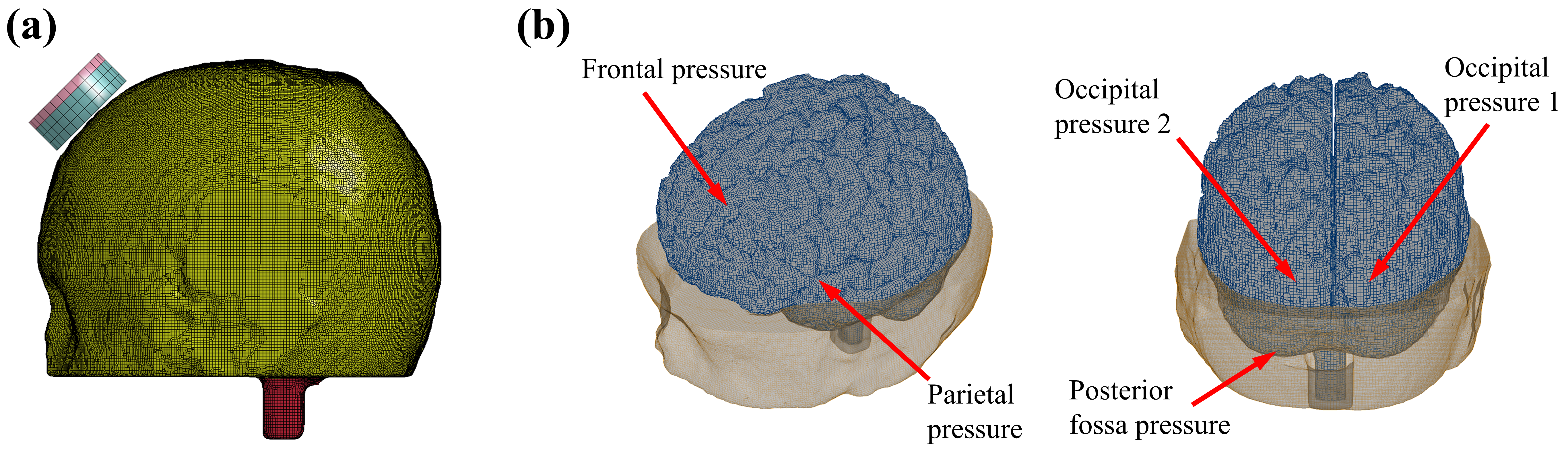


Figure S1 (a) Simulation condition to replicate the Nahum’s experiment (Nahum et al., 1977). (b) Location of pressure measurements in simulation for comparison with Nahum’s experimental data.


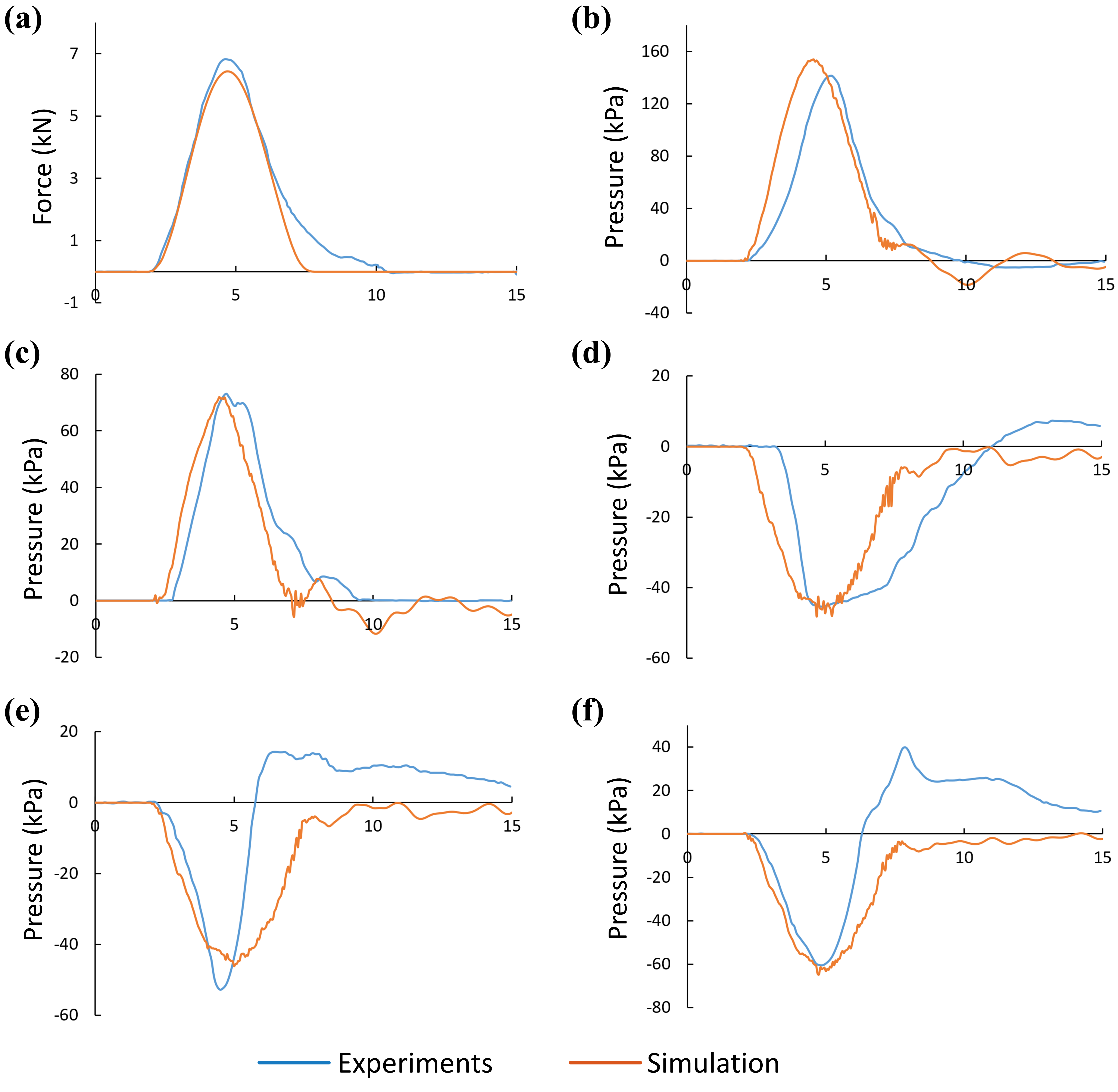


Figure S2 Comparison of (a) impact force time histories, (b) ICP at frontal region, (c) ICP at parietal, (d) ICP at occipital region 1, (e) ICP at occipital region 2 and (f) ICP at posterior fossa region.

**Reference**

Ghajari, M., Hellyer, P.J., and Sharp, D.J. (2017). Computational modelling of traumatic brain injury predicts the location of chronic traumatic encephalopathy pathology. *Brain* 140**,** 333-343.

Nahum, A.M., Smith, R., and Ward, C.C. (1977). "Intracranial pressure dynamics during head impact". SAE Technical Paper).
